# Multi-Scale Feature Fusion Enhancement for Underwater Object Detection

**DOI:** 10.3390/s24227201

**Published:** 2024-11-11

**Authors:** Zhanhao Xiao, Zhenpeng Li, Huihui Li, Mengting Li, Xiaoyong Liu, Yinying Kong

**Affiliations:** 1 School of Computer Science, Guangdong Polytechnic Normal University, Guangzhou 510665, China; xiaozhanhao@gpnu.edu.cn (Z.X.); lizhenpeng@gpnu.edu.cn (Z.L.); lihh@gpnu.edu.cn (H.L.); 2 Guangdong Provincial Key Laboratory of Intellectual Property and Big Data, Guangdong Polytechnic Normal University, Guangzhou 510665, China; 3 School of Data Science and Engineering, Guangdong Polytechnic Normal University, Heyuan 517583, China; limengting@gpnu.edu.cn; 4 School of Statistics and Mathematics, Guangdong University of Finance & Economics, Guangzhou 510320, China

**Keywords:** underwater object detection, DETR, cross-scale feature fusion

## Abstract

Underwater object detection (UOD) presents substantial challenges due to the complex visual conditions and the physical properties of light in underwater environments. Small aquatic creatures often congregate in large groups, further complicating the task. To address these challenges, we develop Aqua-DETR, a tailored end-to-end framework for UOD. Our method includes an align-split network to enhance multi-scale feature interaction and fusion for small object identification and a distinction enhancement module using various attention mechanisms to improve ambiguous object identification. Experimental results on four challenging datasets demonstrate that Aqua-DETR outperforms most existing state-of-the-art methods in the UOD task, validating its effectiveness and robustness.

## 1. Introduction

Nowadays, the utilization of marine ecological resources has predominantly shifted from relying solely on human diving operations to employing remotely operated vehicles and autonomous underwater vehicles equipped with underwater cameras, as referenced in [1]. Imagery underwater object detection (UOD) is crucial for these technologies to effectively perceive and interact with the underwater environment, enabling informed decision-making. With the rapid advancement of artificial intelligence, these devices have been evolving to be more intelligent and capable, facilitating a range of marine tasks, such as underwater garbage collection, ecological monitoring, and wreck exploration [2].

**Figure 1:**
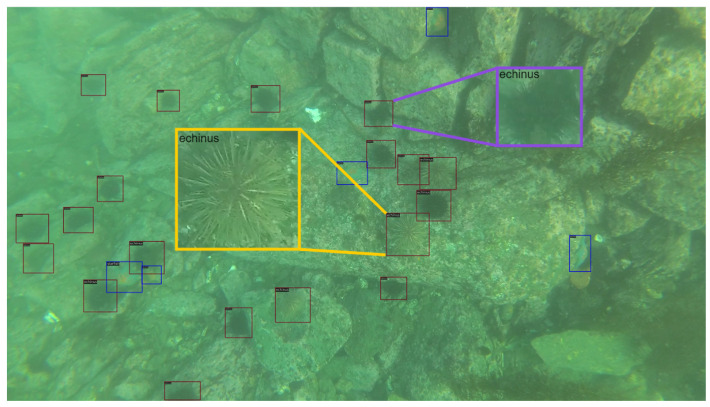
Due to the unavoidable environmental factors such as lighting and water, these aquatic organisms exhibit colors and textures that closely resemble the background. Coupled with low contrast or blurred imagery, they become ambiguous to detect, such as the echinus in the yellow box that presents the similar texture with the rock and the one in the purple box that conceals itself in the crevice. Furthermore, underwater organisms often occupy small areas within the images, such as echini and starfish, aggravating the detection difficulty.

Due to the more complicated underwater environments, generic object detection (GOD) approaches indeed perform unsatisfactorily [3]. In water, light is easy to scatter and be absorbed; underwater images thus often suffer from degradation issues of reduced contrast, color distortion, and fuzziness. The images with low quality make it difficult for object detectors, even for humans, to accurately detect objects from the background [4]. Also, a great number of aquatic creatives have a camouflage habitus to make their texture close to the surroundings, which brings more difficulties to the task. As illustrated in Figure 1, the echinus in the yellow box exhibits a similar texture with the background, and the one in the purple box conceals itself in the crevices, which are ambiguous objects. The minimal visual distinction between ambiguous objects and their surrounding backgrounds complicates the extraction of discriminative and subtle semantic features for these objects. Consequently, this challenge diminishes the accuracy in identifying ambiguous targets. For that, researchers have conducted extensive explorations in recent years. For example, ROIMIX [5] uses data augmentation to simulate occluded and blurred objects, thereby improving performance on such objects. GCC-Net [6] employs an underwater image enhancement model to improve the detection accuracy in complex underwater environments. Dai et al. [7] developed a sophisticated network, ERL-Net, to make clearer differentiation from the background by extracting edge information of underwater objects [7]. However, most existing methods incorporate a cross-scale information exchange module without fully considering the characteristics and relationships of features across different layers. This oversight limits their ability to effectively distinguish ambiguous objects from the background.

Moreover, a common phenomenon that exists in the wild is the clustering of small creatures in large groups. In underwater imagery, the majority of objects typically occupy very small areas. As shown in Figure 1, most of the echinus and starfish only occupy small areas of the image. It leads detectors to lack sufficient information to capture these small and clustered objects and to learn their feature representation. Unfortunately, the degraded quality of underwater images exacerbates this issue. Consequently, the prevalence of small and ambiguous objects in underwater images continues to pose significant challenges for underwater object detection.

In this paper, we propose an end-to-end underwater object detection framework based on Transformer called Aqua-DETR, for ambiguous and small objects. To address the shortcomings of Transformer-based detectors in small object detection in the underwater environment, we design an align-split network (ASN). The network aligns the features in different layers extracted by the backbone network to the same layer, which can interact with each other fully. After the interaction, the features are split and processed to the subsequent network for further fusion. In addition, leveraging cross-attention, channel attention, and graph attention mechanisms, we design a distinction enhancement module (DEM). The module injects detailed appearance information into deep semantic features to find ambiguous objects. Finally, we conduct experiments on four challenging datasets, comparing our method against other state-of-the-art detectors. The experimental results, including ablation analysis, demonstrate the superior performance and generalizability of the proposed framework across various datasets.

The contributions of this paper are summarized as follows:

- We introduce an end-to-end real-time Transformer-based detection framework for underwater small and ambiguous objects.
- We design an align-split network (ASN) for identifying small objects via reinforcing multi-scale feature interaction and fusion. We also develop a distinction enhancement module based on different attention mechanisms to enhance the sensitivity of the model on ambiguous objects.
- The experiment results on four challenging underwater datasets, DUO, Brackish, TranshCan, and WPBB, demonstrate that our method outperforms most other object detection methods in underwater environments.

The rest of the paper is organized as follows: Section 2 reviews related work in underwater object detection. Section 3 provides a detailed description of the proposed method. Section 4 gives the experiments conducted and analysis of the proposed method. Finally, the concluding section summarizes the findings and contributions of this work.

## 2. Related Works

In recent years, advancements in marine robotics have opened up new avenues for ocean exploration. Coupled with advanced machine vision techniques, marine robots demonstrate significant potential for exploring the underwater environment. Underwater object detection plays a pivotal role in ocean exploration by identifying objects in digital images, which provides essential information for subsequent tasks [8]. Driven by deep learning techniques, a rapid evolution of image-based object detection techniques has sprung up over the past two decades [9]. Generic object detection approaches have proven effective in many atmospheric scenarios. However, compared to the atmospheric environment, the underwater environment is more complicated. Images captured in the water often suffer from the quality degradation issue due to the absorption and scattering properties of light within the aquatic environment [10]. Consequently, most generic object detection methods fall short of underwater images, and many researchers have turned their eyes to underwater object detection. Most existing underwater object detection approaches are based on neural network architectures, and they can be categorized into two groups: CNN-based approaches and Transformer-based ones.

### 2.1. CNN-Based Methods

Convolutional neural networks (CNNs) are a class of neural networks that have been used in various image processing tasks. These networks operate by conducting convolution operations through a moving window mechanism, coupled with subsequent pooling operations, which collectively introduce an inductive bias. This approach capitalizes on local spatial correlations within the image data, enabling CNNs to extract features with both effectiveness and efficiency. R-CNN [11] has been the pioneering CNN-based approach for object detection, which divides the task into two stages. Specifically, it first generates region proposals, which is a set of candidate objects, which further classifies and localizes these candidate objects. R-CNN has yielded a series of powerful variants, such as Faster R-CNN [12] and Mask R-CNN [13]. Due to their stable and generic performance, they have been widely adopted in underwater object detection [14]. For instance, ROIMIX [5], an enhancement of Faster R-CNN, introduces a data augmentation technique to simulate overlapping, occluded, and blurred objects. Subsequently, Qi et al. introduced USTD [15], a two-stage R-CNN-like detector incorporating a Deformable Convolutional Pyramid module to address deformation and occlusion challenges. Additionally, Boosting R-CNN [16] was developed to extract more precise region proposals and to emphasize challenging examples through reweighting. Recently, Li et al. [17] improved the feature pyramid network by incorporating a regression strategy based on a tailored cornerness loss to find more accurate bounding boxes. However, the above-mentioned detectors, which are based on the R-CNN framework, often face efficiency challenges and may not meet the real-time requirements for underwater object detection. In contrast, the design of our method, such as the selection alignment layer, meticulously balances prediction accuracy with computational efficiency.

More and more researchers have focused on single-stage CNN-based approaches to address the limitations of the aforementioned methods. These methods directly predict the localization and category of objects to improve the reasoning time. In 2016, the initial YOLO [18] was proposed, which stands out as a real-time framework for its exceptional balance between speed and accuracy. Due to its success in object detection, YOLO has developed into the tenth generation [19]. The YOLO family has also given rise to several variants specifically tailored for the underwater environment. For example, TC-YOLO integrates adaptive histogram equalization and optimal transport, incorporating transformer self-attention and coordinate attention into YOLOv5s to enhance feature extraction for underwater objects [20]. Zhao et al. [21] proposed YOLOv7-CHS, which adopts three novel modules in the detection network to address the color deviation and low illumination issues in harsh underwater conditions [21]. In addition, Shen et al. [22] improved YOLO with a multiple information perception-based attention module for low-quality underwater images. However, most YOLO variants for UOD are anchor-based and require hand-designed components such as non-maximum suppression or anchor generation. The design of these components typically requires prior knowledge about the application scenarios, which limits their generalizability, as discussed in [23]. In contrast, our method is designed as a one-stage, anchor-free approach, thereby requiring fewer hand-designed components and enhancing its versatility.

### 2.2. Transformer-Based Methods

Transformers originally developed in the field of natural language processing and have significantly advanced computer vision in recent years. These methods divide images into non-overlapping patches, which are then linearly projected into token sequences. The self-attention mechanism is applied to these sequences to learn the relationships among patches. Unlike CNNs, which primarily focus on capturing local information, Transformers excel at capturing global context within images, as discussed in [24]. For instance, Dai et al. [6] introduced GCC-Net, which integrates a real-time image enhancement method. It initially generates an enhanced image and then employs the Swin-Transformer architecture to facilitate interaction between the original and the augmented image [6]. Later, Gao, et al. [25] improved CSWin-Transformer and devised a local path detection information scheme to enhance the semantic representation of distinctive features of small-scale underwater objects [25]. Besides, the detection transformer (DETR) [26], which is a one-stage and anchor-free method, has garnered considerable research interest in recent years. It views the object detection task as a direct set prediction problem and utilizes the Hungarian algorithm to assign predicted bounding boxes with ground truth objects. Based on this matching strategy, DETR is a completely end-to-end detection framework, which differs from the CNN-based approaches that require anchor generation and post-processing. Because of its strategy, more and more variants of DETR have been proposed, such as deformable DETR [27] and Co-DETR [28]. In many scenarios of object detection, DETR approaches have achieved state-of-the-art performance. However, a notable drawback of most DETR-like methods is their high computational cost. To address this issue, the real-time detection transformer framework (RT-DETR) was developed, incorporating an efficient hybrid encoder and an uncertainty-minimal query selection scheme, enabling real-time and high-quality detection, as detailed in [29]. The aforementioned approaches primarily concentrate on generic objects, neglecting ambiguous objects concealed within the background. In contrast, our framework is specifically designed to differentiate such objects from their backgrounds.

## 3. Methodology

### 3.1. Model Overview

**Figure 2:**
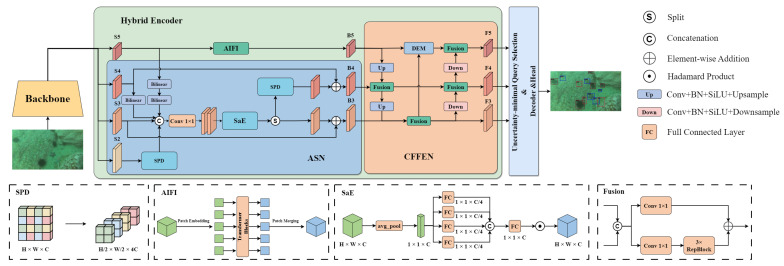
The overview of Aqua-DETR. It takes the feature maps of the last four stages (S2, S3, S4, S5) from the backbone network as the input to the hybrid encoder. The hybrid encoder contains two main networks: the align-split network (ASN) and the cross-scale feature fusion enhancement network (CFFEN). Then the features processed by these networks are fed as object queries to a query selection module, followed by the decoder and the prediction head. Finally, the head predicts bounding boxes of objects in the image and their corresponding categories.

The architecture of Aqua-DETR is depicted in Figure 2. Similar to RT-DETR, Aqua-DETR comprises three principal components: a backbone network, a hybrid encoder, and a Transformer-based decoder with auxiliary prediction heads. In Aqua-DETR, the feature maps in different stages of the backbone network actually capture semantic information across various layers. To effectively detect more small objects without ignoring larger ones, we design an align-split network in the hybrid encoder. This network aligns multi-scale features from different layers, facilitating their interaction and fusion, and then splits them into two distinct layers. Next, the output features are further fused in the following network, the cross-scale fusion enhancement network. In the network, we design a distinction enhancement module to incorporate the detailed appearance features into the deep semantic information for ambiguous objects. Subsequently, the encoder features are processed through an uncertainty-minimal query selection module, which filters out low-quality object queries. Ultimately, utilizing a decoder in conjunction with an inference head, the filtered object queries are transformed into bounding boxes along with their corresponding categories.

### 3.2. Align-Split Network (ASN)

As mentioned above, in the natural underwater environment, commonly, creatives are small and clustered in groups, which brings a big challenge to image-based UOD. In an underwater image, small objects generally occupy limited areas, which are easy to be misunderstood as noise by detectors. On the other hand, when it comes to a close-up image, small objects occupy more areas. It suggests taking a balance among objects of different sizes. For that, we design an align-split mechanism to better understand features in different scales and develop an align-split network (ASN) in the hybrid encoder.

The visual features of an image are extracted by a backbone network layer by layer, and the feature maps of each layer are denoted by S2, S3, S4, and S5 in sequence. Generally, the shallow layer outputs larger feature maps that capture low-dimensional texture details and the positions of smaller objects. In other words, to better identify underwater small objects, it is advisable to take the features of shallow layers, such as S2 or S3, into account. However, larger feature maps mean a higher computation burden, and we need to take a balance between accuracy and efficiency. Inspired by Gold-YOLO [30], we collect S2, S3, S4, and S5 feature maps and align them into the size of S3 via upsampling or downsampling to acquire higher-resolution features and preserve small object details at the same time. Specifically, to minimize the information loss of small objects during downsampling, we employ the space-to-depth (SPD) operation [31] to reduce the feature map $S2\in R^{H\times W\times C}$ to align with $S3\in R^{\frac{H}{2}\times \frac{W}{2}\times C}$. As shown in Figure 2, the SPD operation first slices out a sequence of sub-feature maps by a factor of 2 from the feature map S2 and then concatenates them along the channel dimension to obtain a new feature map $R^{\frac{H}{2}\times \frac{W}{2}\times 4C}$ of the same height and width with S3. On the other hand, feature maps S4 and S5 are resized through interpolation-based upsampling to match the dimensions of S3. So far, these feature maps have the same width and height. Then we concatenate them in channel dimension and obtain $S_{A}\in R^{\frac{H}{2}\times \frac{W}{2}\times 7C}$. The motivation to align the feature maps with S3 is to reduce the computational burden and preserve small object information as possible. It can be formulated as follows:

(1) $$S_{A}=\mathrm{Concat}\left[{\mathrm{Up}}^{2}\left(S5\right),\mathrm{Up}\left(S4\right),S3,\mathrm{SPD}\left(S2\right)\right].$$

where Up means upsampling with a factor of 2 and ${\mathrm{Up}}^{2}$ means a factor of 4.

Then, by applying a 1×1 convolution, $S_{A}$ is fed into Squeeze Aggregated Excitation (SaE) [32] module to get a better fusion representation. The SaE module combines the squeeze and excitation operation in SENet [33] with dense layers to capture channel patterns and learn a global representation. As illustrated in Figure 2, after average pooling, the input feature is linearized and then divided into four branches by fully connected layers. The outputs from the four branches are concatenated and then forwarded to a fully connected layer, which is finally applied with Hadamard production to restore a feature map $S_{F}$ with the same sizes as the aligned feature map $S_{A}$. This process can be denoted as follows:

(2) $$S_{F}=\mathrm{SaE}\left({\mathrm{Conv}}_{1\times 1}\left(S_{A}\right)\right),$$

Subsequently, the feature map $S_{F}$ is split in the channel dimension into $S3^{\prime }\in R^{\frac{H}{2}\times \frac{W}{2}\times C}$ and $S4^{\prime }\in R^{\frac{H}{4}\times \frac{W}{4}\times C}$. To take full advantage of the features extracted by the backbone network, we inject S3 and S4 into the respective branch by element-wise addition operation, and we obtain two feature maps B3 and B4. To align $S4^{\prime }$ with S4, we also adopt the SPD downsampling operation by a scale factor of 2. The formulization is shown as follows:

(3) $$S3^{\prime },S4^{\prime }=\mathrm{Split}\left(S_{F}\right),$$

(4) $$B3=S3^{\prime }⊕S3,$$

(5) $$B4={\mathrm{Conv}}_{1\times 1}\left(\mathrm{SPD}\left(S4^{\prime }\right)\right)⊕S4$$

where ⊕ is the element-wise addition.

As the smallest feature map S5 carries the deepest semantic information, we employ the attention-based intra-scale feature interaction (AIFI) operation [29] on it to better capture the relationships between conceptual entities in the image. After the operation, we can obtain a feature map of the same size, which is designated as B5. Thus, the formula is shown as follows:

(6) B5=AIFI(S5).

In ASM, we adopt an align and split mechanism to aggregate multi-level features by complementing two larger features, which facilitates the detection of smaller objects without reducing the ability to detect bigger objects.

### 3.3. Cross-Scale Feature Fusion Enhancement Network (CFFEN)

After ASM, we employ a cross-scale fusion enhancement network (CFFEN) to further fuse the features across various levels and a distinction enhancement module (DEM) to improve the distinguishment between objects and backgrounds. Inspired by the feature pyramid network (FPN) [34] and path aggregation network (PAN) [35], in CFFEN we adopt a top-down path strategy to fuse cross-scale features and a bottom-up path strategy to augment semantic information from the shallow layers. As shown in Figure 2, a feature fusion path from B5 to B3 is implemented in a top-down manner via convolution, upsampling, and the fusion block. After feature fusion, an opposite fusion path is implemented where the fused features in the shallow layer are used to augment the features in deeper layers. To connect these two paths, the fusion block and the DEM are employed. Specifically, the fusion block consists of two 1×1 convolution and three Repblocks [36].

While the graph convolution network (GCN) and the graph attention network (GAT) have demonstrated success in various computer vision tasks, as evidenced in [37,38], we have developed a distinction enhancement module that builds upon GCN and GAT. By constructing the relation between regions in an image as a graph structure, the features that are not close are related, which helps to find more ambiguous objects.

**Figure 3:**
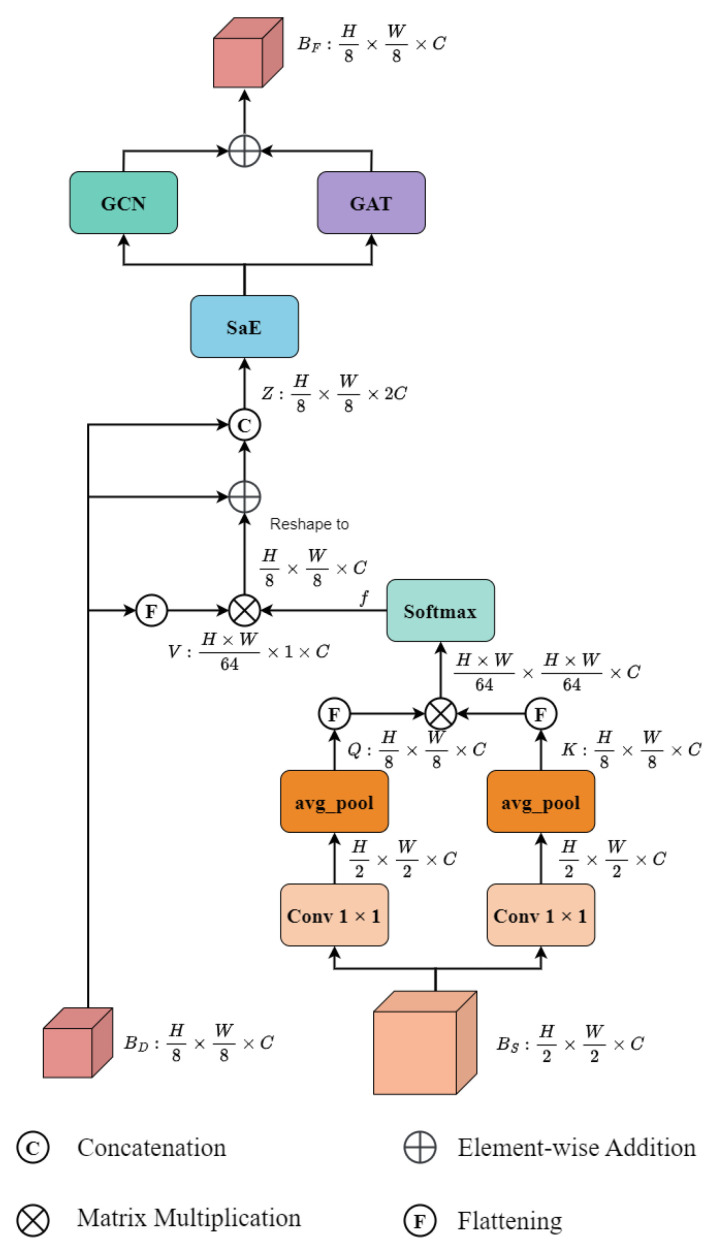
The illustration of the Distinction Enhancement Module (DEM). It takes two feature maps $B_{D}$ and $B_{S}$ as input. In the attention mechanism, $B_{S}$ generates a query *Q* and a key *K*, while $B_{D}$ generates a value *V*. Their multiplication, coupled with a residual of $B_{D}$, to generate a feature map *Z*. Subsequently, it is applied by a channel-wise SaE, followed by GCN and GAT respectively. Finally, these two branches are combined via element-wise addition to produce a feature map $B_{F}$.

In CFFEN, we design a distinction enhancement module based on graph structures to inject detailed appearance features into the deep semantic feature map. Initially, DEM takes a shallow feature map $B_{S}$ and a deep feature map $B_{D}$ as input. Figure 3 illustrates the architecture of DEM. For the shallow feature map $B_{S}$, we first employ two independent 1×1 convolutions with average pooling to obtain two reduced feature maps *Q* and *K*. After flattening and element-wise multiplication operations, we employ a softmax function on them to obtain a correlation attention map *f*. Formally, they can be denoted as

(7) Q=Avgpool(Conv(B3)),

(8) K=Avgpool(Conv(B3)),

(9) f=Softmax(Flatten(Q)⊗Flatten(K)),

To inject the shallow features into the deep feature map $B_{D}$, we multiply the attention map *f* with the flattened feature map $B_{D}$. Subsequently, the resulting feature map is reshaped to the same size as $B_{D}$ and added with $B_{D}$ as a residual connection. Next we concatenate it with $B_{D}$ and call the resulting feature map $Z\in R^{\frac{H}{8}\times \frac{W}{8}\times 2C}$. It is formulated as follows:

(10) V=Flatten(B5).

(11) Z=Concat[B5,B5⊕(V⊗f)].

where ⊗ is the inner product.

Subsequently, we forward *Z* into the SaE module to optimize it in terms of channels to get a global representation. Following this, the output features of the SaE module undergo two branches to take advantage of spatial features: the graph convolution network (GCN) and the graph attention network (GAT) [39]. In these two networks, features are projected into vertices, and the edges among them capture their spatial relations. Those features with similar information are aggregated to reconstruct connections, even though they are distributed sparsely in the space. It helps us to generate more discriminative features to detect ambiguous objects. The ramification of GCN and GAT offers two perspectives to explore high-order semantic relations between image patches and identify discriminative features. As a result, we employ an element-wise addition on their output and obtain a resulting feature map $B_{f}$. Formally,

(12) $$B_{F}=\mathrm{GCN}\left(\mathrm{SaE}\left(Z\right)\right)⊕\mathrm{GAT}\left(\mathrm{SaE}\left(Z\right)\right).$$

The output feature map $B_{F}$ carries deep semantic information with detailed appearance information and is fused into the bottom-up path of CFFEN. Finally, the feature maps F3,F4, and F5 output by the hybrid encoder are concatenated to generation object queries.

### 3.4. Decoder

As the hybrid encoder would generate numbers of object queries, we adopt the uncertainty-minimal query selection module in RT-DETR to optimize the epistemic uncertainty of the encoder features to filter out low-quality queries. Intuitively, the object queries whose predicted distributions of localization differ significantly from those of classification would not be forwarded to the decoder. It helps to reduce computational cost and memory consumption. Ultimately, the decoder with auxiliary prediction heads predicts the categories and bounding boxes of objects from the filtered object queries.

In addition to the loss function of the uncertainty-minimal query selection module, we employ the L1 loss and GIOU loss [40] for box regression and the focal loss [41] for classification, following other DETR-style detectors.

## 4. Experiments

### 4.1. Datasets

To evaluate the performance of our method, we conducted experiments on four underwater object detection datasets: DUO [42], Brackish [43], TrashCan [44], and WPBB [45].

The DUO dataset [42] is derived from the re-annotation of UDD [46] and multiple versions of datasets from the Underwater Robot Professional Contest (URPC). It comprises a total of 7782 images, divided into 6671 for training and 1111 for testing, with 74,515 annotated objects. The dataset includes four categories of underwater objects: holothurian, echinus, scallop, and starfish. Specifically, there are 7887 holothurian objects, 50,156 echinus objects, 1924 scallop objects, and 14,548 starfish objects.

The Brackish dataset [43] is a publicly available underwater dataset containing annotated image sequences of fish, crabs, and starfish in brackish water, with varying levels of visibility. It consists of 14,518 frames with 25,613 annotations randomly split into the training set of 9967 images, the validation set of 1239 images, and the testing set of 1238 images.

The TrashCan dataset [44] comprises a total of 7212 images of underwater trash, including observations of trash, remotely operated vehicles, and diverse undersea flora and fauna, which contains 22 categories. The dataset is divided into 6008 training images and 1204 testing images.

The WPBB dataset [45] focuses on the detection of in-water plastic bags and bottles. It includes 900 fully annotated images, with 500 ones of bags and 400 ones of bottles, split into 720 training images and 180 test images.

### 4.2. Implementation Details

We implemented the proposed approach based on the backbone network Resnet50, utilizing the open-source MMDetection toolbox [47]. We conducted our experiments in the Ubuntu 22.04 system with PyTorch 2.0.1, CUDA 11.3, and Python 3.10. Our method was trained on a single NVIDIA RTX A6000 GPU with 48 GB of memory. Following GCC-Net [6], we use a total batch size of 4 for training. The training schedule for all datasets was set to 36 epochs. We followed the same settings of the hyperparameters in RT-DETR [29]. Optimization was performed using AdamW, with an initial learning rate of 0.0001 and a weight decay of 0.0001. Traditional random flip and resize techniques were applied for data augmentation during training. All other hyperparameters were set as the default settings in the MMDetection toolbox.

### 4.3. Evaluation Metrics

In this paper, we evaluate our method using the standard COCO-format metrics (average precision, AP) [48], which includes AP, ${\mathrm{AP}}_{50}$, and ${\mathrm{AP}}_{75}$. AP is the mean average precision (mAP) calculated by averaging across various thresholds of Intersection over Union (IoU), ranging from 0.5 to 0.95 with an incremental step of 0.05, while ${\mathrm{AP}}_{50}$ is mAP at IoU = 0.50 and ${\mathrm{AP}}_{75}$ is mAP at IoU = 0.75. We also pay attention to object sizes. Specifically, ${\mathrm{AP}}_{S}$, ${\mathrm{AP}}_{M}$, and ${\mathrm{AP}}_{L}$ mean the AP for objects whose area is smaller than 32 × 32, between 32 × 32 and 96 × 96, larger than 96 × 96, respectively. For the efficiency evaluation, we adopt three metrics: Param., FLOPs, and FPS. Param. is the parameter of a detector. FLOPs means floating-point operations per second, and FPS means the number of frames per second.

### 4.4. State-of-the-Art Comparison

We compare our method, Aqua-DETR, against other state-of-the-art methods in the four datasets.

We categorize the detectors into two classes: generic object detectors and underwater object detectors. The first class comprises detectors that are directly applied to the UOD task without any modifications. This class includes generic two-stage detectors like Faster R-CNN [12] and Cascade R-CNN [49], a real-time single-stage detector such as YOLOv7 [50], and two single-stage anchor-free approaches, Deformable DETR [27] and AutoAssign [51].

The second class consists of detectors specifically designed for the UOD task. These detectors optimize various components and training strategies to address issues unique to underwater images, such as data augmentation (ROIMIX [5]), ROI feature enhancement (RoIAttn [52] and Boosting R-CNN [16]), training paradigms (SWIPENet [53]), and the integration of attention mechanisms (ERL-Net [7]). Additionally, GCC-Net [6], YOLOX-S with UnitModule [54], and DJL-Net [51] incorporate underwater image enhancement techniques to mitigate issues with degraded image quality. Our method focuses on enhancing the features of the images and introduces an end-to-end training paradigm tailored for the UOD task. We compare with the methods, which are mainly trained in 36 epochs.

#### 4.4.1. Results on DUO

In Table 1, Aqua-DETR achieves 69.3% in AP, 87.6% in ${\mathrm{AP}}_{50}$, and 76.4% in ${\mathrm{AP}}_{75}$, outperforming most of the approaches in Table 1. Specifically, our method surpasses the renowned generic object detector YOLOv7 [50] by 3.0% in AP. When comparing against the state-of-the-art underwater object detector GCC-Net [6], Aqua-DETR outperforms it by 0.2% in AP but loses by 0.2% in ${\mathrm{AP}}_{50}$. Moreover, Aqua-DETR performs 0.1% in ${\mathrm{AP}}_{75}$ higher than GCC-Net [6], indicating that our method generates more high-quality bounding boxes to locate underwater objects.

#### 4.4.2. Results on Brackish, TrashCan and WPBB

In addition, we validate the superior performance of our method on the Brackish, TrashCan, and WPBB datasets. The experimental results are presented in Table 2. In the Brackish dataset, Aqua-DETR achieves an average precision (AP) of 82.8% and an ${\mathrm{AP}}_{50}$ of 98.9%, outperforming all other methods in terms of ${\mathrm{AP}}_{50}$. It also ranks second in AP, only slightly below the ERL-Net model [7]. The Brackish dataset was collected in brackish water, which is of high turbidity. This results in images with low contrast and a hazy appearance. It is important to note that some of the approaches, such as GCC-Net, employ an underwater image enhancement algorithm as part of the data preprocessing stage. While our method still performs well in the blurry scenario even without the help of image enhancement.

For the TrashCan dataset, our method achieves 42.9% in AP and 63.0% in ${\mathrm{AP}}_{50}$, which are both the top performance. Compared with other datasets, TrashCan has the most categories to be detected. The excellent performance of Aqua-DETR demonstrates that our method is robust in multi-class underwater detection tasks.

In the WPBB dataset, our method outperforms all other methods, with 83.8% in AP and 100.0% in ${\mathrm{AP}}_{50}$, demonstrating that Aqua-DETR can generate more reliable predictions than any other detectors.

Notably, both TrashCan and WPBB include various types of small underwater objects, such as debris. The top performance of our method indicates its excellent potential in detecting small underwater objects. The results on these three datasets also demonstrate that our method has outstanding performance and generalizability in various underwater environments.

In particular, our method achieves performance comparable to that of GCC-Net in the DUO dataset while outperforming it in these other datasets. The reason for this difference lies in GCC-Net’s utilization of an underwater image enhancement model, which is particularly effective for the blue or green color distributions prevalent in the DUO dataset. In contrast, images in other datasets often exhibit gray or blackish tones, which may have reduced the efficacy of the enhancement model. It also demonstrates the robustness of our method.

### 4.5. Ablation Studies

We conducted ablation experiments on DUO and Brackish to verify the effectiveness of our proposed components. We remove the align-split network and the distinction enhancement module from the Aqua-DETR to obtain the baseline where B4=S4 and B3=S3. For ablations, we add the ASN and DEM into the baseline to get comparison methods, denoted as baseline + ASN and baseline + DEM, respectively.

#### 4.5.1. Analysis of the Align-Split Network

The ablation study results of ASN on DUO are presented in Table 3. It can be observed that the baseline with ASN has improvements of 0.6%, 0.5%, and 0.3% in AP, ${\mathrm{AP}}_{50}$, and ${\mathrm{AP}}_{75}$, respectively. It also increases all AP scores across all categories and sizes. In particular, the ASN brings a rise of 2.3% in ${\mathrm{AP}}_{S}$, which demonstrates its effectiveness in detecting small objects. Generally speaking, the ASN effectively enhances the detection accuracy of small objects in the underwater environment.

#### 4.5.2. Analysis of the Distinction Enhancement Module

To verify the effectiveness of the distinction enhancement module, we conducted ablation experiments in both datasets as well.

On the DUO dataset, the DEM leads improvements of 0.2% collectively in AP, ${\mathrm{AP}}_{50}$, and ${\mathrm{AP}}_{75}$. It primarily enhances the detection for echinus and scallops, with increases of 0.4% and 0.5%, respectively. In the underwater images, echinus and scallops exhibit colors and textures that closely resemble those of rocks and seaweed, enabling them to blend seamlessly with the background. Additionally, the peculiarities of light transmission and water quality further contribute to the ambiguity of these objects. The DEM enhances the ability to discern these ambiguous objects. Based on the baseline with the ASN, the DEM achieves better performances in AP and ${\mathrm{AP}}_{50}$ than other ablated methods.

**Figure 4:**
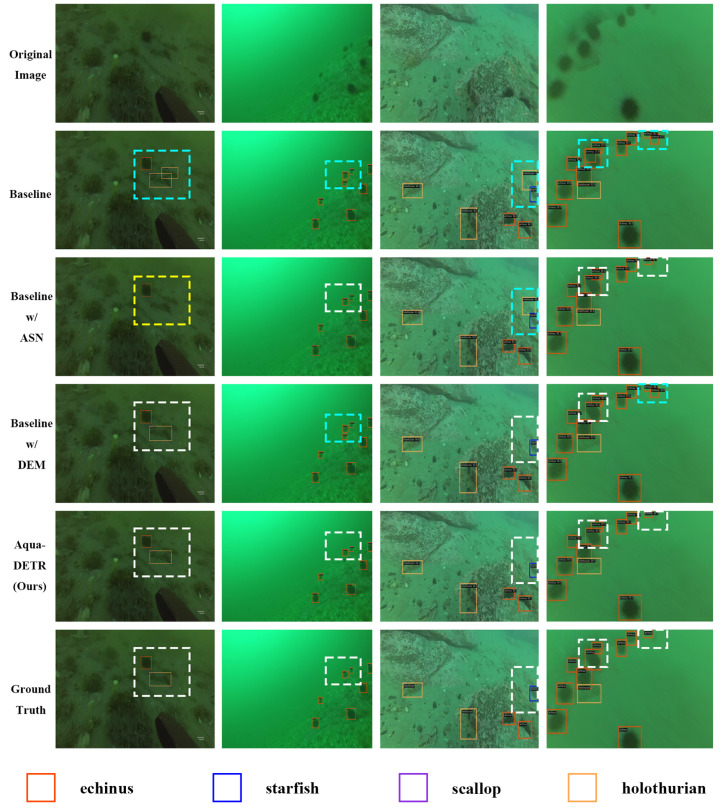
Visual comparison via ablation experiments is presented, showcasing detection results for the baseline, ground truth, and subsequent integration of different components into the baseline model. The white dashed line box indicates the correct detection, while the yellow one indicates the missed detection, and the cyan one indicates the redundant detection.

To further illustrate the effectiveness of the two components, we visualize the prediction results from the test set, as presented in Figure 4. It is evident that the baseline model and its variant with the DEM occasionally misclassify rocks in the background as holothurians or echini. In contrast, the baseline with the ASN tends to more frequently miss the detection of ambiguous objects. The combination of the two components, Aqua-DETR, accurately locates objects with high prediction scores.

### 4.6. Model Complexity Analysis

Next, we analyze the model complexity using the following metrics: model parameters (Params), floating-point operations per second (FLOPs), and frames per second (FPS). The results are presented in Table 4. To keep the same configurations with other detectors, we conduct our method on the DUO dataset with a single GeForce RTX 3090 GPU. The FPS results were tested with a batch size of 1.

As shown in the table, our method attains the second-best inference efficiency, with a rate of 35 in FPS. Only the early generic approach Faster R-CNN beats our method on the inference speed. However, such an early detector is a generic approach and exhibits unsatisfactory performance in the underwater environment. For other metrics, Aqua-DETR operates with 50.36 million parameters and attains 78.33 billion floating-point operations (FLOPs). Although our method does not excel in terms of model metrics, it still outperforms most of the other methods. The design of the proposed components is a balanced outcome, considering both computation latency and prediction accuracy. We contend that our method fulfills the requirements for efficient computing and real-time execution, making it suitable for a wide range of real-world underwater tasks.

### 4.7. Error Analysis

**Figure 5:**
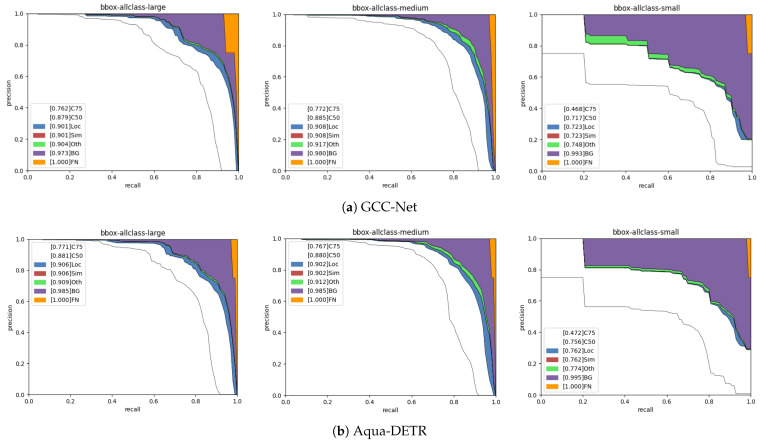
Error analysis plots of the state-of-the-art method GCC-Net (top row) and the proposed method Aqua-DETR (bottom row) across three categories, on the large-sized objects (the first column), medium-sized objects (the second column), and small-sized objects (the last column), conducted on the DUO dataset. As defined in the COCO dataset [48], a series of precision–recall curves with different evaluation settings is shown in each sub-image plot.

We utilize the COCO error analysis tool provided by MMDetection [47] toolbox to conduct a detailed analysis of our proposed method on the DUO dataset. It contains several AP metrics under different conditions. Specifically, C75 and C50 indicate the overall AP under the conditions of the strict IoU = 0.75 and the PASCAL IoU = 0.50, respectively. The Loc and Oth metrics are the overall AP, which removes location errors and classification errors, respectively. The BG metric is the overall AP after removing all background and class false positives, while the FN metric is the AP after removing all the remaining errors. We then compare Aqua-DETR with one of the state-of-the-art methods, GCC-Net, in detecting large-scale, medium-scale, and small-scale objects. Figure 5 shows the results. The plots in each sub-image represent a series of precision–recall curves with various evaluation settings.

In the first column, for the large-scale objects, Aqua-DETR obtains 77.1% and 88.1% in both at a strict evaluation metric IoU = 0.75 (i.e., C75) and the PASCAL evaluation metric IoU = 0.50 (i.e., C50), surpassing GCC-Net by 0.9% and 0.2%, respectively. Disregarding either location errors (Loc) or class confusion (Oth), our method, Aqua-DETR, achieves an improvement of 0.5% over GCC-Net. Furthermore, by eliminating false positives associated with background and class confusion (BG), Aqua-DETR reaches an AP of 98.5%, surpassing GCC-Net [6] by 1.2%. Our method performs better in detecting large-sized objects, which suggests that it does not prioritize small objects at the expense of losing the ability to detect large ones.

For the medium scale, Aqua-DETR achieves 98.5% in BG, which is 0.5% higher than GCC-Net, but performs slightly worse than GCC-Net in other metrics. A possible reason should be that the image enhancement module of GCC-Net contributes to the identification of medium-scale objects.

For the small scale, Aqua-DETR achieves 75.6%, 76.2%, and 77.4% in C50, Loc, and Oth, respectively, outperforming GCC-Net by significant margins of 3.9%, 3.9%, and 2.6%. Compared to larger objects, small objects are more sensitive to correctly detect, as small perturbations in bounding boxes may lead to significant drops in IoU. Our method achieves notably higher results in the Loc metric, demonstrating superior performance on locating small objects.

### 4.8. Qualitative Results

**Figure 6:**
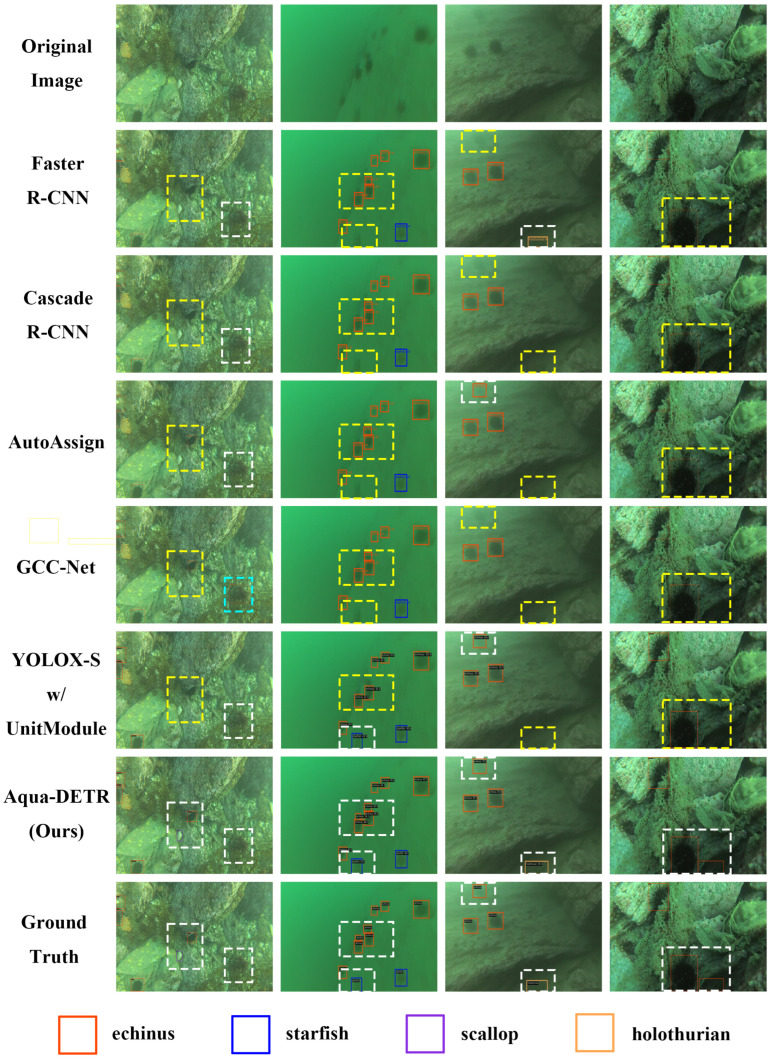
Visual comparison results of different object detection methods on the DUO dataset. Best viewed in color and with zoom. The white dashed line box indicates the correct detection, while the yellow one indicates the missed detection, and the cyan one indicates the redundant detection.

Next, we present several predicted examples of our method and several state-of-the-art methods. Figure 6 shows the visualization of prediction results of the compared methods in DUO. Each column indicates the prediction of the same input. It is not difficult to observe that our method detects more small or ambiguous objects compared to other state-of-the-art methods. In the first column, our method successfully detects a small scallop nestled within the crevice between two rocks. This result aligns with the excellent performance of our model on AP in the scallop category and highlights its strength in identifying small-scale objects. Also, GCC-Net misclassifies a piece of sea-plant as an echinus. In the second column, despite the degraded image quality, our method accurately localizes all instances of echinus. In contrast, other methods exhibit a notable degree of missed detections. In the final two columns, other methods struggle to distinguish ambiguous objects, such as the holothurian that blends with the dusky hues and the echinus situated in the dark area. Our approach, however, excels in differentiating these objects from their backgrounds, even when they share high visual similarities.

**Figure 7:**
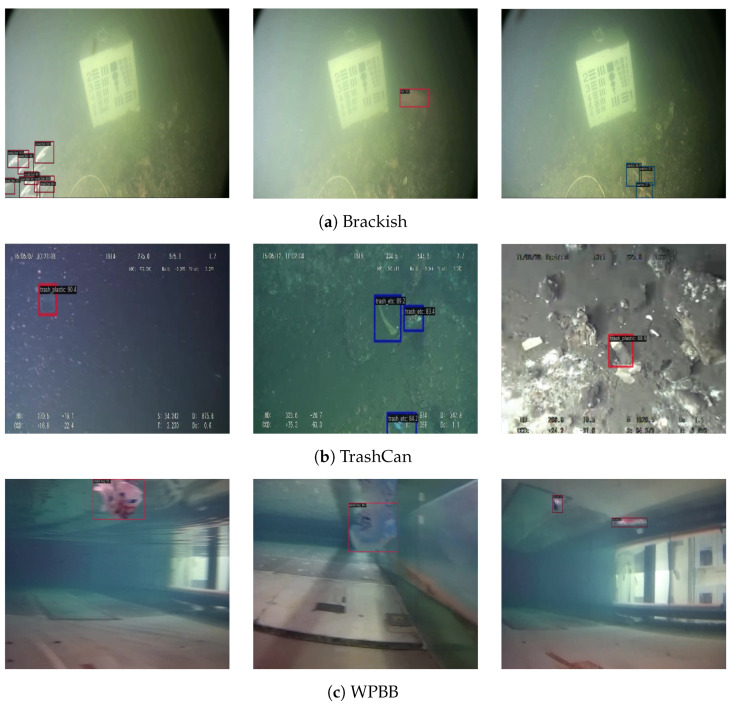
Visualization results of Aqua-DETR on the Brackish, TrashCan, and WPBB datasets. Best viewed in color and with zoom.

Figure 7 illustrates the visualization results of our proposed method on the Brackish, TrashCan, and WPBB datasets. In Brackish, even in the hazy condition, our method effectively locates organisms in the images, which cluster together densely. In TrashCan, although the images experience interference from the low contrast and low-light conditions, our method still successfully detects and correctly classifies the objects. In WPBB, despite the presence of plastic bags with blurred edges, our method provides high-quality bounding boxes for the objects. It is worth noting that these three datasets were collected from different waters and have different object category numbers and distributions. The promising results demonstrate the generalizability and robustness of our method across various underwater environments.

Indeed, the proposed DEM module demonstrates only a modest enhancement in accuracy. However, the integration of ASN and DEM outperforms the use of either individually, contributing significantly to the overall accuracy improvement. One possible reason is that the features provided by ASN, encompassing deep semantic information and shallow appearance characteristics, are more informative, aiding DEM in identifying distinctive features and enhancing their representation. In fact, DEM still has room to be improved. As shown in Figure 2, to keep the efficiency, DEM is only used to may be insufficient for the identification of distinctive features, as it does not incorporate the mid-level feature map. We contend that a more substantial improvement could be achieved by optimizing the architecture of the encoder in a better way.

## 5. Conclusions

In this work, we introduce an end-to-end underwater object detector, Aqua-DETR, to tackle the challenges of small objects and ambiguous objects in underwater object detection. To address these challenges, we propose an align-split network and a distinction enhancement module, which are integrated into the hybrid encoder. The align-split network enhances the feature extraction capability for small objects and maintains a balance among objects in different sizes. The distinction enhancement module effectively incorporates detailed appearance features into deep semantic information, ultimately aiding in the identification of ambiguous objects. We compare the proposed method with various popular object methods, including generic object detectors and underwater object detectors on four challenging datasets. The experimental results demonstrate that our method outperforms most detectors in underwater object detection. Furthermore, the real-time and end-to-end nature of our method makes it highly promising for performing real-world underwater tasks, such as trash cleanup and early fault detection.

Imagery underwater object detection still faces numerous challenges, such as degraded optical images. This is due to the unavoidable presence of complex underwater environments, which prevents us from restoring image quality to levels comparable to those in atmospheric environments. One promising avenue is to incorporate additional modal information, such as sonar images, which should not be difficult to obtain. We believe it is promising to leverage a transformer architecture for multi-modal fusion, thereby reducing the impact of underwater noise and enhancing our object detection capabilities.

## Figures and Tables

**Table 1:** Comparisons with other state-of-the-art methods on the DUO dataset. The results highlighted in red and blue indicate the best and second-best outcomes in each column, respectively.

Method	Year	Epochs	AP	${\mathrm{AP}}_{50}$	${\mathrm{AP}}_{75}$	Echinus	Starfish	Holothurian	Scallop
**Generic Object Detector:**									
Faster R-CNN [12]	2015	36	61.3	81.9	69.5	70.4	71.4	61.4	41.9
Cascade R-CNN [49]	2017	36	61.2	82.1	69.2	69.0	72.0	61.9	41.9
Deformable DETR [27]	2020	36	63.7	84.4	71.9	71.6	73.9	63.0	46.3
AutoAssign [55]	2020	36	66.1	85.7	72.6	74.1	75.5	65.8	48.9
YOLOv7 [50]	2022	36	66.3	85.8	73.9	73.7	74.5	66.3	50.8
**Underwater Object Detector:**									
ROIMIX [5]	2020	36	61.9	81.3	69.9	70.7	72.4	63.0	41.7
RoIAttn [56]	2022	36	62.3	82.8	71.4	70.6	72.6	63.4	42.5
SWIPENet [53]	2022	36	63.0	79.7	72.5	68.5	73.6	64.0	45.9
Boosting R-CNN [16]	2023	36	63.5	78.5	71.1	69.0	74.5	63.8	46.8
GCC-Net [6]	2023	36	69.1	87.8	76.3	75.2	76.7	68.2	56.3
ERL-Net [7]	2024	36	64.9	82.4	73.2	71.0	74.8	67.2	46.5
YOLOX-S w/UnitModule [54]	2024	100	63.7	85.8	72.2	-	-	-	-
DJL-Net [51]	2024	12	65.6	84.2	73.0	-	-	-	-
Aqua-DETR (Ours)	2024	36	69.3	87.6	76.4	74.5	75.4	67.3	58.3

**Table 2:** Comparisons with other state-of-the-art methods on the Brackish, TrashCan, WPBB datasets. The results highlighted in red and blue indicate the best and second-best outcomes in each column, respectively.

Method	Years	Brackish		TrashCan		WPBB	
		AP	AP_50_	AP	AP_50_	AP	AP_50_
Faster R-CNN [12]	2015	61.2	62.9	31.2	55.3	75.7	98.7
Deformable DETR [27]	2020	77.5	97.1	36.1	56.9	73.2	98.3
YOLOv7 [50]	2022	57.2	88.5	24.1	43.4	78.7	99.5
RoIAttn [56]	2022	78.3	91.0	32.6	57.2	70.2	88.1
Boosting R-CNN [16]	2023	79.6	97.4	36.8	57.6	78.5	97.1
GCC-Net [6]	2023	80.5	98.3	41.3	61.2	81.0	99.5
ERL-Net [7]	2024	85.4	98.8	37.0	58.9	79.7	98.5
Aqua-DETR (Ours)	2024	82.8	98.9	42.9	63.0	83.8	100.0

**Table 3:** Ablation studies of the proposed components in Aqua-DETR on the DUO dataset. The bold and red results indicate the best performance of each column. The metrics ${\mathrm{AP}}_{S}$, ${\mathrm{AP}}_{M}$, and ${\mathrm{AP}}_{L}$ are the AP of small (area smaller than 32 × 32), medium (area between 32 × 32 and 96 × 96) and large (area larger than 96 × 96) objects, respectively.

Method	AP	${\mathrm{AP}}_{50}$	${\mathrm{AP}}_{75}$	${\mathrm{AP}}_{S}$	${\mathrm{AP}}_{M}$	${\mathrm{AP}}_{L}$	Echinus	Starfish	Holothurian	Scallop
baseline	68.6	87.0	76.2	50.6	70.1	68.0	74.1	75.5	67.3	57.8
baseline + ASN	69.2	87.5	**76.5**	52.9	**70.7**	68.5	**74.7**	76.0	67.4	**59.1**
baseline + DEM	68.8	87.2	76.4	50.5	70.4	68.0	74.5	75.4	67.3	58.3
Aqua-DETR	**69.3**	**87.6**	76.4	**54.2**	70.5	**68.9**	74.6	**76.3**	**68.0**	58.4

**Table 4:** Model complexity and inference speed of different methods. The results highlighted in red and blue indicate the best and second-best outcomes in each column, respectively.

Method	Backbone	Params	FLOPs	FPS
**Generic Object Detector:**				
Faster R-CNN [12]	ResNet50	41.17 M	63.29 G	41.2
Cascade R-CNN [49]	ResNet50	68.94 M	91.06 G	32.5
Deformable DETR [27]	ResNet50	39.83 M	51.06 G	25.7
**Underwater Object Detector:**				
RoIMix [5]	ResNet50	68.94 M	91.08 G	14.1
Boosting R-CNN [16]	ResNet50	45.95 M	54.71 G	34.7
ERL-Net [7]	SiEdge-ResNet50	218.83M	416.63 G	12.7
RoIAttn [56]	ResNet50	55.23 M	331.39 G	13.1
GCC-Net [6]	SwinFusionTransformer	38.31 M	78.93 G	21.3
DJL-Net [51]	ResNet50	58.48 M	69.51 G	23.2
Aqua-DETR (Ours)	ResNet50	50.36 M	78.33 G	35.0

## Data Availability

The data presented in this study are available on request from the corresponding author.
